# Hepatitis E vaccine—Illuminating the barriers to use

**DOI:** 10.1371/journal.pntd.0010969

**Published:** 2023-01-05

**Authors:** Julia A. Lynch, Jacqueline Kyungah Lim, Philomena E. Peter Asaga, T. Anh Wartel, Melanie Marti, Beno Yakubu, Helen Rees, Kawsar Talaat, Brittany Kmush, Rakesh Aggarwal, Iza Ciglenecki, Emily Gurley, Alain B. Labrique

**Affiliations:** 1 International Vaccine Institute, Seoul, Korea; 2 Department of Immunization, Vaccines and Biologicals, World Health Organization, Geneva, Switzerland; 3 National Agency for Food and Drug Administration and Control (NAFDAC), Nigeria; 4 Wits RHI, University of the Witwatersrand, Johannesburg, South Africa; 5 Department of International Health, Bloomberg School of Public Health, Johns Hopkins University, Baltimore, Maryland, United States of America; 6 Department of Public Health, Syracuse University, Syracuse, New York, United States of America; 7 Department of Gastroenterology, Jawaharlal Institute of Postgraduate Medical Education and Research, Puducherry, India; 8 Médecins sans Frontières, Geneva, Switzerland; 9 Department of Epidemiology, Bloomberg School of Public Health, Johns Hopkins University, Baltimore, Maryland, United States of America; Faculty of Science, Ain Shams University (ASU), EGYPT

## Introduction

Hepatitis E is likely the most common cause of acute viral hepatic disease globally. It is estimated that over 900 million people have been infected with hepatitis E virus (HEV), equivalent to nearly 1 in 8 individuals worldwide [1]. Available evidence suggests that 20 million people are infected annually resulting in over 70,000 deaths [2]. Considering the limited availability of diagnostics, insufficient surveillance, and investigation of hepatitis outbreaks, these are likely significant underestimates.

Although there is only a single HEV serotype, 4 different genotypes have been recognized that commonly infect humans. Genotypes 1 and 2 (G1, G2) are transmitted through sewage contaminated water and are the predominant strains found in large-scale outbreaks in settings with poor access to safe water and sanitation, including in humanitarian emergencies [2–4]. Infections with genotypes 3 and 4 (G3, G4) are mostly sporadic and zoonotic in nature [5] and are believed to be transmitted through consumption of meat in raw forms or direct contact with infected animals; these occur in both low- and high-income settings [6,7]. With an average incubation period of 40 days, outbreaks can be prolonged and may transition into endemic circulation. Blood transfusion is also a recognized route of acquisition, especially in developed regions [8,9], though not exclusively [10].

Some populations are recognized as having higher risk for severe disease and death including pregnant women, immunosuppressed individuals, and persons with preexisting chronic liver disease [11–14]. The outcomes for pregnant women with genotype 1 or 2 HEV infection can be notably severe in both outbreak settings and where infection is endemic; however, data on this relationship with pregnancy and genotype 3 or 4 are lacking. While case fatality in the general population ranges from 0.1% to 4%, 10% to 40% of pregnant women with severe HEV disease can die [14–17]. The manifestations of severe disease include fulminant hepatic failure, hepatic encephalopathy, coagulopathy, and death with mortality rates being the highest in the third trimester [14,18–22]. Further, HEV can result in poor fetal outcomes including preterm labor, fetal distress, and death, both in utero and in the early neonatal period [23]. The risk of symptomatic disease appears to be lower in children; however, children, including infants, can experience severe disease [13,24].

A highly efficacious vaccine has existed for over a decade, Hecolin (manufactured by Xiamen Innovax Biotech Co., Ltd), which has been registered in China (2011) and more recently in Pakistan. The vaccine has not yet been submitted by the company to be reviewed for pre-qualification by the World Health Organization (WHO). Proof of efficacy was demonstrated in a Phase III double-blind randomized clinical trial enrolling 112,604 healthy adults aged 16 to 65 years, using a 3-dose regimen (0, 1, and 6 months). The alum adjuvanted vaccine was found to be safe and demonstrated 100% efficacy in the per-protocol analysis at 1 year and 93% at 54 months [25]. In addition, antibody persistence has been demonstrated out to 6.5 years among a majority of subjects in a subset of Phase III trial participants and declines more slowly in vaccinated subjects as compared to those with natural infection [26]. Of note, the efficacy after 2 doses (0, 1 month) during the 5-month interval before the third dose was also 100% [25].

In May 2015, WHO published a hepatitis E vaccine position paper [27] based on advice from WHO’s Scientific Advisory Group of Experts (SAGE) on Immunization. They acknowledged the significant public health problem posed by hepatitis E, particularly among special populations such as pregnant women and individuals living in displaced persons’ camps. The SAGE further considered Hecolin as a promising vaccine. However, it noted that there were significant data gaps concerning the incidence of HEV infection and disease worldwide, which prevented a recommendation for its routine use. It therefore recommended that national authorities may consider and decide on the use of this vaccine based on their local epidemiology, particularly in certain high-risk situations, such as outbreaks. The WHO suggested specific areas of additional study for Hecolin, which included pregnant women, immunization of individuals under 16 years of age, the elderly, immunocompromised persons, and those with chronic liver disease. In addition, the paper suggested that evaluation of vaccine impact in outbreak situations would be valuable, as would be exploration of alternative and abbreviated dosing schedules more suitable for reactive use during outbreaks and concomitant use with other vaccines. Finally, despite there being only 1 HEV serotype, WHO noted that vaccine efficacy in the Phase III trial had only been shown against genotype 4.

Following the publication of the WHO position paper, several clinical trials have been conducted to expand clinical experience with Hecolin in different populations and address some evidence gaps. In the same time period since 2015, large outbreaks of HEV have occurred resulting in potentially preventable morbidity and mortality [28–32], yet the vaccine has for the first time been implemented as a public health tool in one outbreak setting outside of China in March 2022 [33]. To better understand the continuing barriers to the use of HEV vaccine, including the impact of remaining evidence gaps identified by WHO, we convened a group of HEV experts and representatives from global normative bodies and national vaccination implementation and regulatory agencies for a series of meetings in August 2021. Below are the summary consensus statements we hope will illuminate the remaining barriers to use (Table 1). We further provide some recommendations from this group for actions to be taken by researchers and the public health community to enable the greatest public health impact of this vaccine.

## Barriers to use

### Use of vaccine in an outbreak

Although the 2015 WHO position paper acknowledged that there may be special situations where HEV vaccine should be used, including outbreaks of hepatitis E, decisions regarding use of vaccine were deferred to local/national authorities. There have been several attempts to use Hecolin in outbreaks brokered through Médecins Sans Frontières (MSF) and coordinated with WHO that were unsuccessful. In this regard, it is important to understand what barriers to use were experienced at the country level.

A common feature of HEV outbreaks is the often-delayed identification of the causative agent, a consequence of limited availability of HEV diagnostics. While late detection may reduce the window of time during which vaccine will have an impact, many outbreaks particularly in Africa are quite prolonged offering extended opportunities to reduce continued morbidity and mortality through vaccination. Yet even after declaration of an outbreak, there remain delays in decision-making regarding the use of vaccine. Public health and regulatory authorities in each country must evaluate the vaccine independently through a national review process. Yet some countries may have gaps in technical capacity and resources, for example, in vaccine manufacturing expertise, to conclude quickly on a review. Whereas if the vaccine were pre-qualified, countries with technical gaps could rely on the WHO review and inspections. Awareness regarding the vaccine has been low among regulators, policymakers, and communities where many recent outbreaks have occurred. This lack of familiarity with Hecolin further limits timely assessments of risk versus benefit, a cycle that is repeated with each outbreak in a different country. To lower this barrier, ***global public health proponents should prospectively conduct preparedness sessions regarding HEV and Hecolin with public health and regulatory leaders in high-risk regions*.**

The WHO Regional Technical Advisory Groups on Immunization (RITAGs) provide an established network that could facilitate dissemination of information and enable more informed and rapid local decision-making during outbreaks. Familiarity with the vaccine could additionally be advanced by conduct of HEV epidemiologic and vaccine studies in populations where outbreaks occur. Such research activities will not only advance knowledge through eventual publications, but also the scientific and ethical review of protocols will engage the local medical, public health, and regulatory bodies and expand awareness in the civil society of HEV burden and consequences, and the existence of the vaccine. Future studies conducted in areas where the disease is most common, such as in South Asia and Africa, could aim to address the remaining vaccine evidence gaps described in this paper, achieving several objectives at once.

Although demonstration of feasibility and acceptability of HEV vaccination in outbreaks will hopefully catalyze broader use, it would be ideal to measure effectiveness of the vaccine in an outbreak setting. Acknowledging the many challenges of conducting research amidst an ongoing outbreak, study designs exist that can be applied in relatively austere outbreak settings. To facilitate implementation of effectiveness studies when vaccine is deployed, implementation agencies (MSF, WHO) have developed generic protocols that can be rapidly adapted to a specific setting. ***Familiarization with these protocols should also be part of the prospective information sessions held with public health and regulatory leaders in high-risk regions*.**

Another barrier that prevented deployment during one outbreak was the lack of vaccine supply. Because demand for HEV vaccines is low, the manufacturer produces the vaccine in relatively small quantities and intermittently. The lead time for additional production can be up to 6 months. Hence, supply for export may be limited if requested to respond to a large outbreak. ***Relevant global bodies should consider advance purchase agreements and/or establishment of a global vaccine stockpile so that supply is available at the time of need*.**

### Preventing disease among pregnant women using vaccination

Pregnant women are at the highest risk for morbidity and mortality with HEV infection. There is an ongoing study in Bangladesh to define the effectiveness of Hecolin administered to women of child-bearing age in preventing HEV-related morbidity when those women subsequently become pregnant [34,35]. In this important study, 20,000 non-pregnant women aged 16 to 39 years in 1 large rural community were enrolled and randomized to receive either Hecolin or an active control (hepatitis B vaccine) and are being followed with 2 years of active community surveillance for hepatitis. However, there are limited data on the use of vaccine in women already pregnant. In the Phase III trial in China, there were 37 women who inadvertently received vaccine while pregnant [36]. The vaccine was well tolerated, and the rate of adverse events in these pregnant women was similar to that in pregnant women who had inadvertently received the placebo. However, nearly half of all pregnancies in both groups were electively terminated limiting the number of evaluable pregnancy outcomes. All women who gave birth had healthy term infants. No immunogenicity data was collected among these women, limiting possible insights available from this serendipitous cohort. Consequently, because of this limited data, the WHO recommendations do not support routine use of vaccine in pregnant women, but do not preclude its use during an outbreak, relying on local risk-benefit judgement.

Perceptions about using vaccines in pregnant women are changing with recommendations for routine use of several vaccines (e.g., tetanus, influenza), and new vaccines specifically targeting pregnancy (e.g., RSV, GBS) are advancing in clinical trials. Other outbreak diseases of high consequence (e.g., Ebola, Coronavirus Disease 2019 [COVID-19]) have challenged public health and regulatory authorities to make risk-benefit assessments of vaccine use in pregnant women with limited data with most countries recommending vaccine use in pregnancy [37]. Hecolin, a non-replicating protein-based vaccine, has little theoretical risk to a mother–fetus pair, and reproductive toxicology studies in animals have suggested no concerns (personal communication). Though there is no indication that Hecolin would pose an increased risk to a pregnancy or would be less immunogenic in pregnant women, ***the risk-benefit decision-making regarding administration of vaccine to pregnant women would be significantly advanced by studies expanding the amount of data on the safety and immunogenicity of Hecolin in pregnant women***. Such studies should consider dosing regimens that do not increase safety risk but are likely to provide a rapid short-term protection that is required for outbreaks. Considering the short-term efficacy of 100% after 2 doses in the Phase III trial, 2 doses administered 1 month apart during pregnancy in a high-risk setting would be a logical approach with the third dose provided after delivery.

### Administration of vaccine to those under 16 years of age

Hecolin is currently licensed for use among individuals ≥16 years of age and there are no data regarding its safety, immunogenicity or efficacy in younger age groups as they have not been included in any trial to date. In outbreaks and endemic settings, disease among children is less common than among adults, though it does occur [24]. The role of children in asymptomatic transmission of HEV is unclear and understudied. Though some cross sectional seroprevalence studies in presumed endemic settings indicate low seroprevalence among children and increasing seropositivity with age, in a cross-sectional serosurvey conducted at the end of a large urban outbreak in Chad, children under 5 years had the highest IgM prevalence at 12.6% that declined to 4.6% for those >15 years [24,38]. Risk factors identified for symptomatic HEV infection included having 2 or more children below the age of 5 in a household [39]. If asymptomatic children are important in transmission, vaccination of younger children could reduce the risk of infection among higher-risk individuals in the population. One modeling study that assumed children are involved in transmission evaluated several vaccination strategies to be applied to an outbreak that included combinations of (1) vaccinate only those non-pregnant and 16 to 65 years (consistent with the vaccine label); (2) include those under 16 years; (3) include or only vaccinate pregnant women. Based on their model, the most effective vaccination strategy in preventing mortality in an outbreak is to use vaccine in all ages including children and include pregnant women [40]. These modeling results are provocative; however, the value of expanding the age indication of Hecolin to children younger than 16 years in an outbreak setting remains theoretical. The potential value would be clearer with the availability of more epidemiologic data regarding the burden of disease among children and their role in transmission.

However, for routine use in endemic settings, an expanded age indication may be critically important to facilitate programmatic introduction in areas with high transmission. New vaccine introductions are least costly and most effective if they can be linked to existing health care delivery programs such as the Expanded Programme on Immunization (EPI) for young children or school-based vaccination programs such as those for HPV vaccine which target 9 to 14 years. Few such programs exist at age 16 or older. How young to administer the vaccine and achieve effect at preventing disease depends in large part on the duration of protection provided by the vaccine. The vaccine developers have demonstrated that high efficacy is retained out to 4.5 years post vaccination. If long-term persistence of protection is demonstrated, expansion of the age indication to within the window of other vaccine and health care delivery programs could be very important. For example, routine vaccination of girls/young women co-incident with delivery of HPV vaccine as they enter reproductive age could prevent morbidity and mortality associated with HEV infection during pregnancy in endemic areas. Considering the potential value to include those under 16 years in reactive vaccinations during outbreaks, and the desirability of leveraging existing vaccine delivery programs for routine use, ***the evaluation of the safety and immunogenicity of Hecolin in those under 16 years should be pursued*. *In addition*, *estimates of vaccine-associated protection beyond 4*.*5 years should be made available*.**

### Demonstration of cross-genotype protection

Though the genotype pairs G1/G2 and G3/G4 appear to have ecological preferences in terms of animal host range, all of them belong to 1 serotype. Cross protection was first established with an earlier G1 capsid-based virus-like particle vaccine that demonstrated cross protection to all genotypes in a primate challenge model [41]. At least 1 highly conserved, universally neutralizing epitope has been identified [42]. A dominant immunogenic site, it is functionally linked to viral adsorption to host cells, and antibodies targeting this site are abundant in naturally infected individuals as well as Hecolin vaccine recipients. Hecolin, based on a G1 virus capsid protein, was protective against a G4 challenge in a primate model; an observation replicated in the Phase III trial in humans [25,43]. An ongoing effectiveness study of Hecolin in Bangladesh may provide new direct evidence in humans of the expected homotypic protection of the G1 vaccine against G1. Given the absence of any data that suggests a lack of cross protection to all genotypes, ***this vaccine should be considered for use against any genotype when disease risk warrants in this single serotype infection*. *Observational studies following real world use of the vaccine will likely provide confirmation of cross genotype protection in humans*.**

### Other special populations

Trials completed since 2015 have demonstrated that Hecolin is safe and immunogenic among those over 65 years [44] and those who are healthy but hepatitis B surface Ag positive [45]. In addition, a study has been completed in individuals with chronic liver disease, but is yet to be published [46]. This is an important and likely common special population in many settings where HEV is transmitted as HEV superinfection could accelerate disease progression and increase the mortality. Another common special population waiting for study would be those immunocompromised by HIV and those with medically induced immune suppression. ***Publication of the study of Hecolin in individuals with chronic liver disease should be expedited to better inform the use of vaccine in this high-risk population*. *Evaluation of the vaccine in special populations should be pursued*, *particularly those that are common or have evidence of high risk for severe illness*.**

### Alternative dosing regimens

The currently approved 3-dose schedule of Hecolin (0, 1, and 6 months) is costly and challenging to deliver in an outbreak setting and among displaced populations where health care infrastructure is limited and follow-up of individuals difficult. In the Phase III clinical trial, the vaccine afforded 100% protection for a 5-month period after the second dose of the vaccine in an endemic setting [25]. Therefore, 2 doses, given 1 month apart, could provide significant short-term protection. What remains unknown is whether even a single dose would be sufficient for short-term impact in an outbreak. Also unknown is whether the third dose is indeed required for longer term protection. The number of doses required for short- or long-term effect may also be impacted by prior HEV infection.

The manufacturer has conducted a study to evaluate the safety and immunogenicity of an accelerated regimen of 0/7/21 days [47]. In this study, the geometric mean concentration of anti-HEV antibody 1 month after the third dose in the accelerated schedule was non-inferior to the standard regimen (0, 1, and 6 months). However, this schedule would be challenging to deliver in an outbreak. Therefore, sufficient data already exists to suggest that ***vaccination during an outbreak could reasonably prioritize delivery of 2 doses as a primary urgent intervention to prevent immediate morbidity and mortality*, *especially among the highest risk groups***. Delivery of the third dose could be accomplished after the emergency with the objective of preventing future events. Clinical endpoint trials to refine dosing regimens are difficult and costly to perform. ***Observational studies following real world use of the vaccine in outbreak and endemic settings are highly encouraged as they will likely provide practical information regarding allowable flexibility in dosing regimens and their durability***.

### Concomitant use

Studies of concomitant use are typically conducted when 2 vaccines are likely to be administered at the same time for programmatic efficiency. With only a few exceptions, seroconversion rates and adverse event frequency are not impacted by simultaneous administration of the most-commonly used live and inactivated vaccines [48–50]. One study has convincingly demonstrated that concomitant administration of Hecolin with another protein-based vaccine, hepatitis B, was both safe and did not adversely affect immune response to either vaccine [51]. While additional studies of the administration of Hecolin with other vaccines likely to be administered at the same time would be helpful for programmatic implementation, it is impractical to expect every possible combination to be evaluated. Therefore, ***the absence of specific concomitant use data should not preclude the use of HEV vaccine in settings where the risk of HEV infection is high and concomitant administration of an additional vaccine provides the potential for greatest benefit to the population*.**

**Table 1:** Summary of barriers to use and recommendations.

Barriers to use	Recommendations
Limited prior awareness regarding HEV and the vaccine among regulators, policymakers, and communities in outbreak-prone regions results in hesitant decision-making in the face of outbreaks	***Global public health proponents should prospectively conduct preparedness sessions regarding HEV and Hecolin with public health and regulatory leaders in high-risk regions***
Although efficacy was established in an endemic region, there has been only 1 use of vaccine in an outbreak	***Familiarization with generic protocols to assess effectiveness in outbreak conditions should be part of the prospective information sessions held with public health and regulatory leaders in high-risk regions so feasibility*, *acceptability*, *and effectiveness can be documented***
There is uncertainty about supply of vaccine when needed	***Relevant global bodies should consider advance purchase agreements and/or establishment of a global vaccine stockpile so that supply is available at the time of need***
Although recombinant protein vaccines have low theoretical risk during pregnancy, there is limited data on safety and immunogenicity of the vaccine in pregnant women	***Studies expanding the amount of data on the safety and immunogenicity of Hecolin in pregnant women should be prioritized in order to advance the risk-benefit decision-making regarding administration of vaccine to pregnant women in outbreaks and endemic settings***
There are no data regarding safety, immunogenicity, or efficacy in younger age groups as they have not been included in any trial to date	***The evaluation of the safety and immunogenicity of Hecolin in those under 16 years should be pursued and estimates of vaccine-associated protection beyond 4*.*5 years should be made available to inform programmatic delivery options***
Both the standard and accelerated dosing regimens are difficult to deliver in an outbreak setting	***Sufficient data already exists to support delivery of 2 doses as a primary urgent intervention to prevent immediate morbidity and mortality*, *especially among the highest risk groups*. *Observational studies following real world use of the vaccine in outbreak and endemic settings are highly encouraged as they will likely provide practical information regarding allowable flexibility in dosing regimens and their durability***
Cross protection to all genotypes has not been demonstrated in humans	***The vaccine should be considered for use to reduce risk of disease for any genotype in this single serotype virus infection***
There is no data on safety or immunogenicity among individuals immunocompromised by HIV or those with medically induced immune suppression	***Evaluation of the vaccine in special populations such as those immunocompromised or with chronic liver disease should be pursued and results made publicly available*, *particularly those that are common or have evidence of high risk for severe illness***
There is limited data on concomitant use with other vaccines	***The absence of specific concomitant use data should not preclude use when the administration of an additional vaccine provides the potential for greatest benefit to the population***

## Conclusion

Although there are gaps in understanding the true burden of HEV globally, existing data are more than adequate to confirm that the prevalence and consequences of HEV infection are significant. The excess mortality and severe disease experienced by vulnerable groups, notably pregnant women, and displaced populations, are deserving of urgent global attention. Given the existence of a safe and highly effective vaccine, it is imperative that we identify and work to remove barriers and bottlenecks to its use in outbreaks and highly endemic settings. We hope that this review and expert opinion will lead to concrete actions by public health authorities and decision-makers, as well as focus research efforts and funding to reduce the unnecessary loss of life and productivity caused by hepatitis E around the globe.

[3–5 Learning Points]

1. Hepatitis E virus (HEV) is one of the most common causes of acute viral hepatitis globally, but the burden is underestimated.
2. There is a highly efficacious vaccine, Hecolin, which was found to be safe and efficacious in a Phase III trial, but WHO SAGE did not recommend its routine use due to the significant lack of data on disease burden and use of vaccine outside of the trial population.
3. In 2021, a series of meetings was organized among HEV experts and representatives from global and national vaccination implementation and regulatory agencies to discuss the existing barriers to the use of hepatitis E vaccine.
4. Some of the barriers identified were: the lack of the technical capacity and resources to conduct a national review process in the absence of WHO pre-qualification for a timely decision-making on use of the vaccine in outbreak settings; the lack of vaccine supply, given the low demand for HEV vaccines and the long lead time for additional production; and the lack of evidence on safety, immunogenicity in younger individuals, pregnant women, and special populations such as those immunocompromised or with chronic liver disease.
5. Some of the recommendations from the group were: further data generation from HEV epidemiologic and vaccine studies to expand clinical data and increase awareness and understanding of the burden to support decision-making; advance purchase agreements and/or establishment of a global vaccine stockpile to be available upon urgent request.

[5 Key Papers in the Field]

1. Li P, Liu J, Li Y, Su J, Ma Z, Bramer WM, et al. The global epidemiology of hepatitis E virus infection: A systematic review and meta-analysis. Liver Int 2020;40(7):1516–28. Epub 2020 Apr 24. doi: doi: 10.1111/liv.14468. PubMed Central PMCID: PMCPMCID: PMC7384095 PMID: 32281721.
2. Cooper BS, White LJ, Siddiqui R. Reactive and pre-emptive vaccination strategies to control hepatitis E infection in emergency and refugee settings: A modelling study. PLoS Negl Trop Dis. 2018;12(9):e0006807.
3. Spina A, Lenglet A, Beversluis D, de Jong M, Vernier L, Spencer C, et al. A large outbreak of Hepatitis E virus genotype 1 infection in an urban setting in Chad likely linked to household level transmission factors, 2016–2017. PLoS ONE. 2017;12(11):e0188240.
4. Zaman K, Dudman S, Stene-Johansen K, Qadri F, Yunus M, Sandbu S, et al. HEV study protocol: design of a cluster-randomised, blinded trial to assess the safety, immunogenicity and effectiveness of the hepatitis E vaccine HEV 239 (Hecolin) in women of childbearing age in rural Bangladesh. BMJ Open. 2020;10(1):e033702.
5. Zhu FC, Zhang J, Zhang XF, Zhou C, Wang ZZ, Huang SJ, et al. Efficacy and safety of a recombinant hepatitis E vaccine in healthy adults: a large-scale, randomised, double-blind placebo-controlled, phase 3 trial. Lancet. 2010;376(9744):895–902.

[Up to 3 advantages and 3 disadvantages of the new technology]

1. (advantage) There is an available vaccine, Hecolin, and it is registered in China and Pakistan.
2. (advantage) In a Phase III double-blind randomized clinical trial, the vaccine was found to be safe and highly efficacious with 93% efficacy at 54 months.

1. (disadvantage) The vaccine has not yet been submitted for pre-qualification by the World Health Organization.
2. (disadvantage) In 2015, WHO published a hepatitis E vaccine position paper based on advice from the Scientific Advisory Group of Experts (SAGE) on Immunization and its routine use was not recommended given the significant lack of data on the incidence of HEV infection and disease.
3. (disadvantage) There is a lack of data on its use and to date, there has been only 1 application of the vaccine by public health authorities in an outbreak setting.

## Disclaimer

The authors alone are responsible for the views expressed in this article and they do not necessarily represent the views, decisions, or policies of the institutions with which they are affiliated.
